# Extrusion-Blown PBAT/Thermoplastic Starch (TPS) Active Films Incorporated with Grape Seed Extract for Extending the Shelf Life of Peanut Butter

**DOI:** 10.3390/foods14234094

**Published:** 2025-11-28

**Authors:** Xiaosong Zhai, Zhen Guo, Limin Zheng, Fei Zhao, Rui Zhang

**Affiliations:** 1 Shandong Facility Horticulture Bioengineering Research Center, Jia Sixie College of Agriculture, Weifang University of Science and Technology, Weifang 262700, China; guozhen_wky@163.com (Z.G.); 18654877088@163.com (L.Z.); feizhaozhaofei@126.com (F.Z.); 2 School of Pharmaceutical Sciences and Food Engineering, Liaocheng University, Liaocheng 252000, China; zhangrui@lcu.edu.cn

**Keywords:** poly(butylene adipate-co-terephthalate), starch, grape seed extract, extrusion blowing, active packaging

## Abstract

In this study, poly(butylene adipate-co-terephthalate) (PBAT), starch, glycerol, and grape seed extract (GSE) were blended and extruded to fabricate PBAT/thermoplastic starch(TPS)/GSE active films by blow molding. The interaction between GSE and TPS primarily occurred through hydrogen bonding, with little interaction observed with PBAT. The oxygen barrier property of the film was improved by the incorporation of GSE into the films, whereas the mechanical properties slightly decreased. The PBAT/TPS/GSE films had excellent UV blocking properties imparted by PBAT and visible light blocking properties endowed by GSE. The films containing GSE offered antimicrobial activity against *Escherichia coli* and *Staphylococcus aureus* by delaying bacterial growth. Also, the GSE-added films exhibited antioxidant activity with strong dose dependence due to the free radical scavenging ability of polyphenolic compounds in GSE. The shelf life of peanut butter packaged with the PBAT/TPS/GSE-5 film was expected to exceed 300 days, which was approximately twice that of LDPE film packaging. The proposed active films had good material properties, functional activities, and excellent ability to prolong the shelf life of peanut butter.

## 1. Introduction

Packaging plays a crucial role in the modern food industry, not only protecting products from external environmental influence, but also extending their shelf life and enhancing their market competitiveness [1]. As public awareness of food safety and environmental protection continues to rise, the development of packaging materials that combine functionality and environmental friendliness has emerged as a research focus [2,3].

Over the past few decades, excessive consumption of non-degradable plastic has resulted in a substantial accumulation of plastic waste in the environment, which has raised great concerns about biodegradable alternatives [4,5]. Current biodegradable polymers such as poly(lactic acid) (PLA), polyhydroxyalkanoates (PHAs), poly(butylene adipate-co-terephthalate) (PBAT), cellulose, starch, etc., are commonly used in many packaging applications due to their biodegradability. PLA is easy to process using standard plastic manufacturing techniques and is compostable, making it a leading candidate for sustainable food packaging materials. PLA exhibits outstanding tensile strength, but its use in food packaging, especially in films, is severely restricted due to its inherent brittleness and limited flexibility [6,7]. PBAT is also an excellent degradable plastic with processing conditions and mechanical strength similar to low-density polyethylene (LDPE). Notably, the elongation at break and flexibility of PBAT are much higher than those of PLA, which leads to the widespread application of PBAT in the manufacturing of packaging films. Nevertheless, some drawbacks such as slow degradation rate, high production cost, and lack of functionality somewhat restrain its comprehensive access to the application of the food packaging industry [8]. Starch is a green and sustainable resource that can be synthesized into thermoplastic starch (TPS) through heating in the presence of plasticizers. TPS has shown great commercial potential in the packaging field due to its sustainability, renewability, low cost, and rapid degradation [9]. Many works have proved that the technology of preparing composite films by blending starch or TPS and PBAT is a promising alternative for producing eco-friendly biodegradable plastics [10,11,12].

The functional packaging film further augments the packaging’s capacity to preserve, improve, or alter the quality and safety of packaged products [13,14]. Recently, some active compounds have been integrated into films as augmenters to endow the packaging films with antioxidant, antimicrobial, or other specific functional properties. These compounds are capable of migrating from the packaging to the packaged food to enhance food safety. Some synthetic additives have potential pollution risks, which is contrary to the original intention of food safety and environmental protection [15,16]. Therefore, the exploration of natural additives from plants has become a solution in recent years. The valorization of agricultural waste or residue into value-added by-products for food packaging represents a sustainable practice, which supports zero-waste objectives and satisfies consumers’ demands for food safety and environmental protection [14]. Some bioactive ingredients have recently been extracted from agricultural waste and applied to food preservation and active packaging [17,18]. For example, rice straw extracts significantly enhanced the antioxidant and antimicrobial properties of PHBV films and extended the shelf life of cold-stored meat [19]. The double-layer film developed using sunflower by-products exhibited synergistic antioxidant and antibacterial properties against *E. coli* and *S. aureus*, and significantly extended the shelf life of chicken breasts [20]. The jackfruit seed starch-based edible films incorporated with pomegranate peel extract exhibited excellent antioxidant activity and prolonged the shelf life of white grapes [21]. In this work, grape seed extract (GSE) was selected as the active augmenter and incorporated into PBAT/TPS films to enhance both antioxidant and antimicrobial activities. Grape (*Vitis vinifera* L.) is one of the most consumed fruits worldwide, whether as fresh table fruits or as processed products such as wine, juice, and sauce, among others. Grape seeds are produced in large quantities as waste by-products in the wine, juice, and sauce industries [4,22]. GSE predominantly involves abundant polyphenolic compounds, such as procyanidins, flavonoids, gallic acid, tocopherol, and epicatechin, all of which are responsible for the antioxidant and antimicrobial activities. In fact, GSE is certified by the US Food and Drug Administration (FDA) as a safe additive [5,23]. At present, GSE has been added to some biopolymers, such as polysaccharide, protein to prepare active films, but most of these studies use the solvent casting which is difficult to scale up production [1,4,23,24]. The most extended technology to obtain film materials is by blending polymers with various additives to form masterbatches from which the packaging films are thermoplastically molded by different technologies such as extrusion, blowing, coating, etc. Melt extrusion blow molding is the preferred method for industrial film production due to its high efficiency and stability. To the best of our knowledge, few works have been conducted on developing PBAT/TPS active films incorporated with GSE by extrusion blowing.

In this study, the PBAT/TPS/GSE films active were continuously and stably prepared by extrusion blowing. The effects of GSE incorporation on the physicochemical properties and functional activities of the blend film were assessed. The functional film was applied as a food packaging material on peanut butter to prolong its shelf life by reducing lipid oxidation.

## 2. Materials and Methods

### 2.1. Materials

PBAT (Biocosafe^TM^ 2003F) was procured from Xinfu Pharmaceutical Co., Ltd. (Hangzhou, China), featuring a density of 1.25 g/cm^3^ and a melting temperature of 115 °C. The molecular weight distribution of the PBAT was <2 × 10^5^ g/mol with a peak value of 5 × 10^4^ g/mol [25]. Modified cassava (*Manihot esculenta* Crantz) starch (HP-CF T0278) was supplied from Hangzhou Starpro Starch Co., Ltd. (Hangzhou, China) with a hydroxypropyl group content of 3.1%. Peanut butter, without the addition of antioxidants, was bought from a local supermarket near Tai’an, China. Glycerol (analytical grade) was purchased from Kaitong Chemical Reagent Co., Ltd. (Tianjin, China). GSE (red-brown powder, polyphenols > 95% and proanthocyanidins > 70%) was provided by Fufeng Sinoute Bio-Tech Co., Ltd. (Xi’an, China). All the other chemicals employed in this study were of analytical grade and utilized as received.

### 2.2. Film Preparation

**Figure 1:**
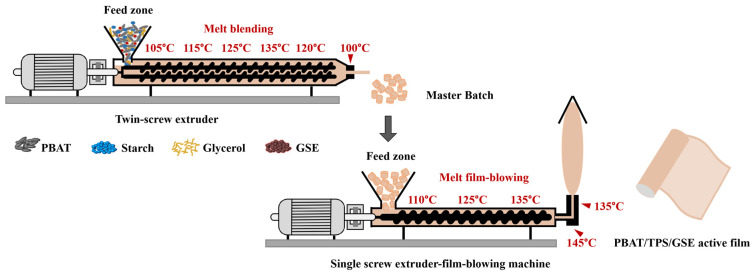
The schematic diagram for the fabrication process of PBAT/TPS/GSE active films.

PBAT (800 g), starch (150 g), glycerol (50 g), and GSE (0, 10, 30, and 50 g) were mixed and then compounded by a twin-screw extruder (SHJ-20B Giant, Nanjing, China). The extruder temperature profile was set to 105–115–125–135–120–100 °C with a screw rotation speed of 170 rpm. The extrudates were air-cooled and then pelletized. Finally, the PBAT/TPS/GSE films were produced using a lab-scale film-blowing machine (SCM-50, Lianjiang, Zhangjiagang, China) at the temperature setting of 110–125–135–145–135 °C and the screw rotation speed of 30 rpm. The PBAT/TPS/GSE films with GSE concentrations of 0, 1, 3, and 5 wt% were coded as PBAT/TPS/GSE-0, PBAT/TPS/GSE-1, PBAT/TPS/GSE-3, and PBAT/TPS/GSE-5, respectively. The schematic diagram for the fabrication process of PBAT/TPS/GSE active films is listed in Figure 1.

### 2.3. Film Characterization

#### 2.3.1. Fourier Transform Infrared Spectroscopy (FTIR)

The FTIR spectra of the PBAT/TPS/GSE films were obtained via a spectrometer equipped with an iD5 ATR sampling accessory (TNicolet iS5, Thermo Fisher Scientific, Waltham, MA, USA). The spectra were recorded within the range from 4000 to 550 cm^−1^, with a resolution of 4 cm^−1^ and 32 scans conducted for each sample.

#### 2.3.2. X-Ray Diffraction (XRD)

The XRD analysis of the PBAT/TPS/GSE films was carried out using an X-ray diffractometer (D8 Advance, Bruker–AXS, Karlsruhe, Germany) within 2θ ranging from 10° to 40° at 2°/min. The relative crystallinity was calculated by dividing the crystalline area by the total area.

#### 2.3.3. Scanning Electron Microscopy (SEM)

The film was cryo-fractured in liquid nitrogen to prepare fracture specimens. All samples were pre-coated with a thin layer of gold, and then the morphologies were taken using a Regulus 8100 SEM (Hitachi, Tokyo, Japan).

### 2.4. Film Properties

#### 2.4.1. Color Measurement

Color measurements were performed by a CR-40 colorimeter (Konica Minolta, Tokyo, Japan), and the color parameters (L*, a*, and b*) were recorded. The total color difference (Δ*E*) was computed by the following equation [2].

$$∆E=\sqrt{{\left(L^{*}-L_{0}^{*}\right)}^{2}+{\left(a^{*}-a_{0}^{*}\right)}^{2}+{\left(b^{*}-b_{0}^{*}\right)}^{2}}$$

#### 2.4.2. Optical Properties

The light transmittance rate of PBAT/TPS/GSE films against UV and visible light was explored by measuring the light transmittance using a UV–Vis spectrophotometer (UV-8000A, Metash, Shanghai, China) at wavelengths of 280 nm (T_280_) and 660 nm (T_660_). The films were cut into rectangles (1 cm × 4 cm) and placed directly into the spectrophotometer with air as a reference. Each film was repeated at least three times.

#### 2.4.3. Thermogravimetric Analysis (TGA)

The PBAT/TPS/GSE films were heated from 25 °C to 600 °C with a heating rate of 10 °C/min using a thermogravimetric analyzer (DTG60A, Shimadzu, Kyoto, Japan) in a nitrogen atmosphere. The change in mass over temperature was recorded as a TG thermogram, and the derivative form of TG (DTG) was obtained by differentiating the TG values.

#### 2.4.4. Mechanical Properties

The films were accurately cut into strips (150 mm × 15 mm), and the film thickness was measured at six stochastic points using a digital micrometer with an accuracy of 0.001 mm. Tensile strength (TS) and elongation at break (EAB) of the films were obtained via an XLW (CP) tensile tester (Labthink, Ji’nan, China) with a pulling speed of 100 mm/min. Each test was repeated at least six times.

#### 2.4.5. Barrier Properties

Oxygen permeability (OP) and water vapor permeability (WVP) of the films were measured using a permeability tester (BTY-B1, Labthink, Ji’nan, China) and an automatic WVP tester (PERME^TM^ W3/030, Labthink, Ji’nan, China) at 38 °C and 90%RH, respectively [8].

### 2.5. Functional Activities of Films

#### 2.5.1. Total Polyphenol Content (TPC)

Ethanolic extract: The ethanolic extract of the films was prepared by submerging 150 mg of film fragments into 10 mL of ethanol in the dark and shaking at 37 °C and 150 rpm for 48 h. The TPC in the PBAT/TPS/GSE films was determined by measuring the TPC in the film extract according to the Folin–Ciocalteu method described by Dash et al. [26] with some modifications. A mixture composed of 0.5 mL of the ethanolic extract, 0.5 mL of Folin–Ciocalteu reagent, 4.5 mL of 7.5% (*w*/*v*) NaCO_3_ solution, and 4.5 mL of deionized water was subjected to thorough oscillation. The resulting solution was reacted in the dark for 30 min, and the absorbance was measured at 760 nm using a microplate reader. Ethanol was used as a blank. The results were expressed as gallic acid equivalent per unit mass of the film (mg gallic acid equivalent/g film), determined by using a Gallic acid standard curve.

#### 2.5.2. Antioxidant Activity

The antioxidant activity of the film was determined using the DPPH and ABTS free radical scavenging methods. For the DPPH free radical scavenging analysis, a methanolic solution of DPPH was prepared by dissolving 4 mg of DPPH in 100 mL of methanol. Subsequently, 5 mg of a sample film was added to 10 mL of the DPPH solution, reacted in the dark for 4 h, and the absorbance was measured at 517 nm. For the ABTS free radical scavenging analysis, 7 mM ABTS was combined with 2.45  mM potassium persulfate (1:1, *v*/*v*) to generate a free radical solution. The solution was kept in the dark for 16 h, and then the absorbance was made 0.7  at 734 nm by diluting the solution. Then, 5 mg of the sample film was added to 10 mL of the ABTS solution, and the absorbance at 734 nm was measured after reacting in the dark for 4 h.

Antioxidant activity for each assay was calculated using the following equation.

$$Antioxidant\;activity\;(\%)=\frac{A_{0}-{\;A}_{s}}{A_{0}}\times 100$$

where *A*_0_ and *A_s_* are the absorbance of the blank and film samples, respectively.

#### 2.5.3. Antimicrobial Activity

One strain of Gram-positive bacteria, *Staphylococcus aureus* (*S. aureus*, CMCC26003), and one strain of Gram-negative bacteria, *Escherichia coli* (*E. coli*, CVCC1387), were employed to assess the antimicrobial activity of the films following the method of Som et al. [27] with some modifications. *E. coli* was provided by the Agricultural Culture Collection of China, and *S. aureus* was maintained in the College of Food Science and Engineering, Shandong Agricultural University.

The bacteria were pre-cultured in liquid medium (Luria–Bertani) at 37 °C for 24 h to prepare exponential growth cultures. Subsequently, these cultures were diluted with a 0.9% (*w*/*v*) sodium chloride solution to obtain a bacterial solution with a concentration of approximately 10^6^ – 10^7^ CFU/mL. The films were punched into standard disks with a diameter of 6 mm. Each disk was UV irradiated for 15 min and placed horizontally in a 10 mL centrifuge tube. 50 μL of bacterial solution was dropped onto the disk surface and subsequently incubated at 37 °C for 3 h. The bacteria were washed with phosphate- buffered saline (PBS) and evenly spread on the agar plate and subsequently incubated at 37 °C for 24 h. The number of viable colonies on the agar plates was then counted. The inhibition efficiency of bacteria was calculated according to the following equation.

$$\mathrm{Inhibition}\;\mathrm{efficiency}\;(\%)=\frac{x-y}{x}$$

where *x* and *y* are the number of colonies from the positive control and film sample, respectively.

### 2.6. Peanut Butter Packaging and Storage Test

The occurrence of off-flavor due to oxidation of food lipids, commonly known as rancidity, is a significant cause of the quality degradation of foods with high lipid content during storage. Peanut butter contains over 40 wt% lipid and is prone to oxidative rancidity over time. In this work, peanut butter (46 wt% lipid) was chosen as the subject for packaging and storage tests to evaluate the efficacy of PBAT/TPS/GSE active films. Briefly, for each test set, 25 g of peanut butter was packaged with the LDPE film (commercially available), PBAT/TPS/GSE-0, and PBAT/TPS/GSE-5 films using a vacuum packaging machine, with the unpackaged ones as controls. All samples were stored at 23 °C. Peroxide and acid values of the roasted peanuts during storage were determined to test the effect of packaging on lipid oxidation prevention.

### 2.7. Statistical Analysis

The data were expressed as the mean ± standard deviation. Statistical significance of each data was analyzed using ANOVA via SPSS 21 (IBM, New York, NY, USA). Comparisons among the mean values were determined using Duncan’s multiple range tests at a 5% significance level.

## 3. Results and Discussion

### 3.1. Morphological and Chemical Structure of the Film

#### 3.1.1. Morphology

**Figure 2:**
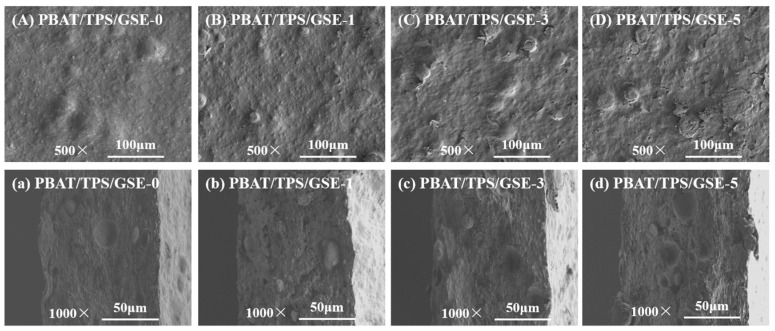
(**A**−**D**) Surface and (**a**−**d**) fracture morphologies of PBAT/TPS/GSE films.

The surface and fracture morphologies of all films are shown in Figure 2. The PBAT/TPS/GSE-0 film exhibited a heterogeneous structure with spherical clumps dispersed throughout the matrix, suggesting the occurrence of immiscibility between TPS and PBAT [13,28]. These domains observed under the microscope seem to be probably unplasticized starch granules. GSE might limit the plasticization of starch granules by hindering the mobility of polymer molecules during processing [29]. Compared with the PBAT/TPS/GSE-0 film, the incorporation of GSE into the films further resulted in rougher morphology and more clumps, and this was intensified with the increase in GSE concentrations. These observations suggested that GSE further aggravated polymer immiscibility and might compromise interfacial adhesion between the matrices. The hydrophilic nature of TPS enhanced its attraction to polar molecules such as GSE, resulting in phase separation within the composite. Leelaphiwat et al. found that the incorporation of nisin and EDTA into PBAT/TPS films also resulted in such a rough microstructure [30]. The formation of these aggregates indicated the deteriorating interfacial interactions between TPS and PBAT, which might damage the mechanical properties of the films [31,32].

#### 3.1.2. FTIR

**Figure 3:**
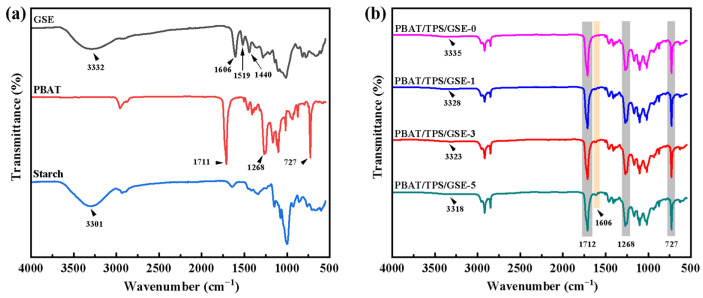
FTIR spectra of (**a**) GSE, PBAT, starch, and (**b**) PBAT/TPS/GSE films.

Figure 3 shows FTIR spectra of PBAT, starch, GSE, and PBAT/TPS/GSE films. The stretch vibration of the phenolic hydroxyl group in the GSE molecule was located at 3332 cm^−1^ [24,33]. The observed peaks at 1606, 1519, and 1440 cm^−1^ may be attributed to the C=C stretching vibration of aromatic rings in GSE molecules [34]. The signal of GSE was observed at 1606 cm^−1^ in the spectrum of the PBAT/TPS/GSE films and gradually became stronger with the increase in GSE concentrations, indicating that GSE was effectively integrated into the films [32,35].

The characteristic absorption peaks of PBAT were observed at 1711 and 1268 cm^−1^ (C=O and C−O stretching vibration of ester carbonyl group) as well as 727 cm^−1^ (C−H out-of-plane bending vibration of the phenyl ring) [36]. The incorporation of GSE into PBAT/TPS films caused no significant alterations in either position or intensity of these characteristic peaks of PBAT. This indicated that the molecular interactions between PBAT and TPS, as well as GSE, were established through physical blending rather than the formation of new covalent bonds. A broad and strong band was detected at 3301 cm^−1^ in the spectra of starch, which was ascribed to O−H stretching vibrations related to intramolecular and intermolecular hydrogen bonding [37,38]. Upon blending starch with PBAT, the PBAT/TPS/GSE-0 film exhibited characteristic peaks corresponding to both starch and PBAT. The plasticization of starch predominantly involves the interaction between plasticizer molecules and the hydroxyl groups of starch through hydrogen bonding, leading to the disruption of hydrogen bonds within the starch molecules [39]. Therefore, compared with the broadband associated with O−H stretching vibration in the starch, the PBAT/TPS/GSE-0 film showed a notable blueshift to 3335 cm^−1^, indicating the decreased average strength of the starch hydrogen bonds [40]. Nevertheless, with the increase in GSE concentrations, the broadband corresponding to the O-H stretching vibration redshifted from 3335 to 3318 cm^−1^, indicating enhanced hydrogen bonding. This might be attributed to GSE serving as a potential anti-plasticizer, hindering the migration of polymer molecules and thereby restricting the plasticization of starch granules [29], as evidenced by SEM images. On the other hand, the interaction between GSE and starch could occur during the preparation process of the GSE-loaded PBAT/TPS/GSE films. This process might promote the formation of new hydrogen bonds between the hydroxyl groups in GSE and starch, resulting in stronger and tighter interactions [35,41]. It was speculated that GSE might preferentially disperse in the TPS phase rather than the PBAT phase due to the similar polarity and strong hydrogen bonding between the two [35]. Tian et al. also verified that a higher content of TPS was advantageous for the combination between cinnamaldehyde and the PBAT/TPS film, as well as the dispersion and retention of cinnamaldehyde within the film [32].

#### 3.1.3. XRD

**Figure 4:**
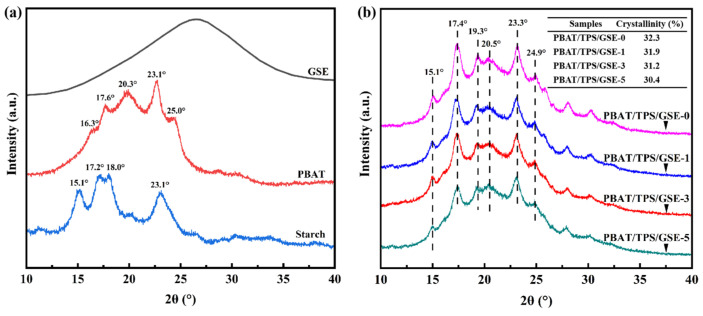
XRD patterns of (**a**) GSE, PBAT, starch, and (**b**) PBAT/TPS/GSE films.

The XRD patterns of all samples are presented in Figure 4. As shown in Figure 4a, a typical amorphous characteristic was observed in the XRD pattern of GSE, with a broad peak around 26.5°. The XRD pattern of starch was dominated by the characteristic peaks at 2θ = 15.1°, 17.2°, 18.0°, and 23.1°, exhibiting a typical A-type crystal structure [42]. The XRD pattern of PBAT was characterized by diffraction peaks at 2θ = 16.3°, 17.6°, 20.3°, 23.1°, and 25.0°, corresponding to the (011), (010), (110), (100), and (111) crystal planes, respectively, representing the *α*-form triclinic crystalline morphology of PBAT [43,44]. As depicted in Figure 4b, after undergoing two thermo extrusion processes, some of the original peaks became indistinct in the XRD patterns of PBAT/TPS/GSE films. This was presumably attributable to the disruption of crystal structures by thermomechanical treatment [45]. The XRD spectra of PBAT/TPS/GSE films with different GSE loadings showed no notable difference, indicating that the addition of GSE had no significant effect on the crystal structure of the films. However, the incorporation of GSE at 1, 3, and 5 wt% led to a slight reduction in the overall crystallinity of the films, from 32.3% (PBAT/TPS/GSE-0) to 31.9%, 31.2%, and 30.4%, respectively. This might be ascribed to the increase in the disorder of the molecular arrangements caused by the presence of GSE in the polymer’s microstructure. As discussed in FTIR, the incorporation of GSE disrupted the original molecular arrangement within the film by establishing hydrogen bonding between GSE and the TPS phase in the film matrix [46]. The coexistence of different crystalline phases without the emergence of new diffraction peaks indicated that no new chemical complex was formed [47].

### 3.2. Film Properties

#### 3.2.1. Film Color and Light Transmittance

Color constitutes a crucial element in food packaging design, which exerts an impact on consumers’ overall acceptance of packaged products [3]. The color parameters of PBAT/TPS/GSE films are summarized in Table 1. When GSE was added, the redness (a*) and yellowness (b*) of the films increased sharply due to the characteristic orange-red color of GSE [1]. Meanwhile, the lightness (L*) of the films decreased significantly by increasing GSE concentrations (*p* < 0.05), suggesting that the increase in GSE concentrations turned the film darker. As reported by Hopkins et al., these extracts could scatter light, thereby creating darker films [48]. As the GSE concentrations increased from 0 to 5%, the total color difference (Δ*E*) increased from 1.13 to 41.30, which indicated a visual change in the surface color of the film caused by the incorporation of GSE. As shown in Table 1, all the films exhibited near-complete UV blocking, which was attributed to the π-electron-rich functional groups inherent in the chemical structure of PBAT, including benzene, benzoic acid, and benzoic acid methyl ester moieties [49]. The incorporation of GSE reduced the transmittance of the film to visible light, which might be ascribed to the impenetrable matrix formed by phenolic interactions, facilitating visible light scattering through the film [50]. Overall, the color of the packaging films can impact consumer acceptability and specific food applications, where darker and less transparent films can provide advantages when packaging light-sensitive foods [51].

#### 3.2.2. Thermal Stability

**Figure 5:**
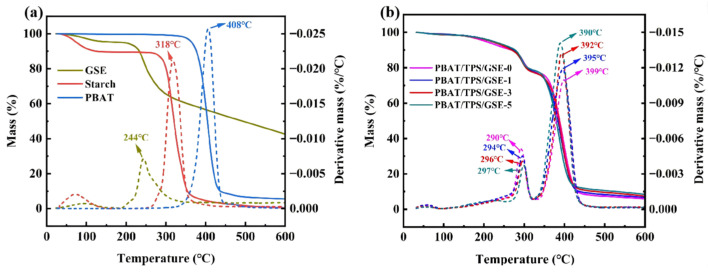
TG and DTG thermograms of (**a**) GSE, PBAT, starch, and (**b**) PBAT/TPS/GSE films.

The thermal stability of PBAT, starch, GSE, and PBAT/TPS/GSE films was assessed by thermogravimetric analysis. The corresponding thermogravimetric (TG) and derivative thermogravimetric (DTG) thermograms are depicted in Figure 5. As shown in Figure 5a, GSE degradation was observed in the range of 190–340 °C, while starch and PBAT exhibited thermal degradation at 260–360 °C and 340–450 °C, respectively. The thermal stability of PBAT/TPS/GSE films, as illustrated in Figure 5b, displayed three degradation stages. The initial stage of mass loss, occurring below 100 °C, was primarily attributable to the evaporation of water from the film, with minimal mass loss attributed to the film’s low water adsorption. Subsequently, the second mass loss occurring at approximately 260–320 °C was the result of the degradation of glycerol and starch. The presence of GSE improved the thermal stability of the TPS phase in PBAT/TPS/GSE films by enhancing hydrogen bonding with starch and inhibiting starch plasticization [52]. The third and major mass loss stage, occurring approximately 260–440 °C, was correlated with the decomposition of the PBAT matrix. The incorporation of GSE into PBAT/TPS/GSE films resulted in reduced thermal stability of the PBAT phase, possibly due to the increase in the disorder of the molecular arrangements caused by the presence of GSE in the polymer’s microstructure, as verified by XRD.

#### 3.2.3. Mechanical Properties

The mechanical properties of the films were evaluated by TS and EAB, as detailed in Table 2. The TS and EAB of the PBAT/TPS/GSE-0 film were 15.82 MPa and 749.33%, respectively. The introduction of GSE resulted in a decrease in TS and EAB, indicating a shift towards decreased flexibility. GSE strongly interacted with TPS and modified the properties of the TPS phase that presented as a dispersed phase in PBAT matrices [53]. This strong interaction potentially weakened the intermolecular adhesion forces within the polymer matrix and exacerbated the phase separation between TPS and PBAT, consequently diminishing TS and EAB of the films [54]. PBAT, serving as the continuous phase, was predominantly responsible for the mechanical properties of the composite films. The incorporation of GSE resulted in many unplasticized starch granules that were dispersed throughout the film matrices, as shown in the SEM image. As heterogeneous structures in the film matrices, they could compromise the inter-substrate densification and integrity of the film matrices, resulting in an uneven distribution of stress under applied force and ultimately reducing the film’s fracture resistance [55,56].

#### 3.2.4. Barrier Properties

The barrier properties of the films were assessed through the measurement of WVP and OP. As depicted in Table 2, the WVP of PBAT/TPS/GSE films exhibited a decline as the GSE concentrations increased, suggesting enhanced water vapor barrier property. The transmission of water vapor through PBAT/TPS/GSE films primarily occurred via the starch phase that contains abundant hydrophilic hydroxyl groups, as water vapor permeates through hydrophilic regions [55]. The observed reduction in WVP of PBAT/TPS/GSE films could be attributed to strong interactions between the hydrophilic hydroxyl groups present in TPS and GSE, potentially restricting the availability of hydroxyl groups in PBAT/TPS/GSE films for interactions with water molecules [52,57]. Oxygen is a diatomic nonpolar molecule. PBAT is composed of a non-polar chain, which allows the passage of non-polar oxygen through the PBAT matrix with little resistance [58,59]. The OP value of PBAT/TPS/GSE films decreased with the increase in GSE concentrations. This trend was ascribed to the polar nature of GSE, which enhanced the oxygen barrier property by slowing down the diffusion of non-polar oxygen through the films [2,60].

### 3.3. Functional Activities

#### 3.3.1. Antioxidant Activity

**Figure 6:**
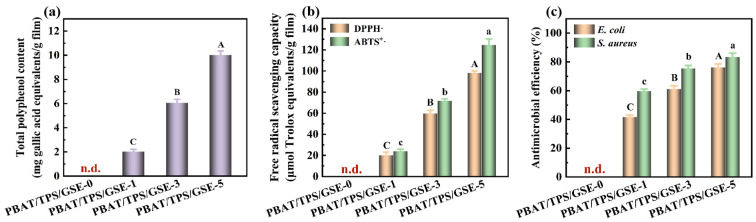
(**a**) Total polyphenols content, (**b**) antioxidant activity, and (**c**) antimicrobial activity of PBAT/TPS/GSE films. n.d. in the figure indicates not detectable. Different letters within the same indicator indicate significant differences among the samples at *p* < 0.05.

GSE serves as an abundant reservoir of polyphenols, encompassing procyanidins, flavonoids, gallic acid, tocopherol, and epicatechin. The TPC of PBAT/TPS/GSE films was determined via the Folin–Ciocalteu method, and the results are presented in Figure 6a. No polyphenols were identified in the PBAT/TPS/GSE-0 film without GSE, whereas the TPC in GSE-loaded PBAT/TPS/GSE films exhibited a significant increase with the increase in GSE concentrations.

The antioxidant activity of PBAT/TPS/GSE films was evaluated by DPPH• and ABTS^+^• scavenging assay, as shown in Figure 6b. The PBAT/TPS/GSE-0 film showed no free radical scavenging capacity, whereas the obvious scavenging capacity was observed in the presence of GSE. The antioxidant capacity of PBAT/TPS/GSE films gradually strengthened with the increase in GSE concentrations. The antioxidant activity of the PBAT/TPS/GSE-3 and PBAT/TPS/GSE-5 films was nearly three times and five times higher than that of the PBAT/TPS/GSE-1 film, indicating a strong dose-dependent relationship. Previous studies have shown a linear correlation between the polyphenol content of extracts and their antioxidant activity [3,5,61,62]. The high antioxidant activity of the GSE-loaded films was attributed to the abundant polyphenols in GSE, particularly proanthocyanidins that possess multiple hydroxyl groups capable of donating hydrogen atoms to neutralize free radicals [3,4,62]. This hydrogen donation results in the formation of stable phenoxyl radicals, effectively interrupting free radical propagation and oxidative degradation pathways [5]. The increase in free radical scavenging capacity with higher GSE concentrations thus reflects the dose-dependent availability of GSE as an antioxidant augmenter.

#### 3.3.2. Antimicrobial Activity

The antimicrobial activity of PBAT/TPS/GSE films with and without GSE was evaluated against *E. coli* (Gram-negative) and *S. aureus* (Gram-positive). The number of viable colonies on the agar plates was quantified to evaluate the antimicrobial efficacy of the films. Films demonstrating a statistically significant reduction in colony counts relative to the positive control were considered to exhibit antimicrobial activity [63]. Figure 6c demonstrated almost no inhibitory effect of the PBAT/TPS/GSE-0 film against *S. aureus* and *E. coli*, confirming that starch and PBAT had no antimicrobial activity. A strong inhibition efficiency was observed with a minimum amount of GSE added into PBAT/TPS films. Only a few colonies survived when exposed to the PBAT/TPS/GSE-5 film. The inhibition efficiency of PBAT/TPS/GSE films against *E. coli* and *S. aureus* significantly increased with the increase in GSE concentrations in the films, suggesting the dose-dependent antimicrobial properties. The polyphenols rich in GSE cause metabolic disorders by disrupting cell permeability, thereby inhibiting bacterial growth or causing bacterial death. The antimicrobial activity assay showed notable inhibition efficiency against both Gram (+) bacteria *S. aureus* and Gram (−) bacteria *E. coli*, with a more pronounced inhibition efficiency observed against *E. coli*. This might be due to the multilayer structure (peptidoglycan layer, lipoprotein layer, phospholipids, or lipopolysaccharide layer) of *E. coli* (Gram-negative bacteria), which causes the cell walls to be more resistant to foreign substances [4]. Alelah et al. indicated that the chitosan/GSE/CuO biocomposite exhibited higher inhibition efficiency against the Gram (+) bacteria *S. aureus* and *S. mutans* than against the Gram (−) bacteria *E. coli* and *K. pneumonia* [62]. Zheng et al. also concluded that Gram (+) bacteria (*S. aureus*) were more susceptible to the pullulan polysaccharide/xanthan gum/GSE active films compared with Gram (−) bacteria (*E. coli* and *B. subtilis*) [23].

### 3.4. Peanut Butter Packaging Application

**Figure 7:**
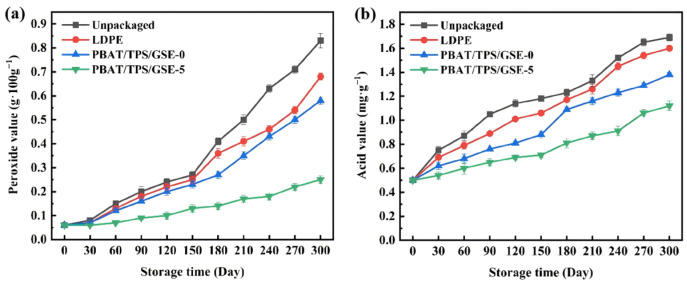
(**a**) Peroxide value and (**b**) acid value changes in peanut butter packaged with the LDPE, PBAT/TPS/GSE-0, and PBAT/TPS/GSE-5 films during storage.

Peanut butter was selected for the packaging application to assess the effectiveness of PBAT/TPS/GSE active films. The main reason for the spoilage of peanut butter is the lipid oxidation caused by its high lipid content. The peroxide value is an important indicator for evaluating initial oxidation, which is associated with the presence of peroxides originating from polyunsaturated fatty acids in lipids [64]. A peroxide value of 0.25 g·100 g^−1^ is a tipping limit for lipid oxidation. Beyond this limit, the edible quality of lipids will rapidly deteriorate. As shown in Figure 7a, the peroxide value of peanut butter gradually increased over time. The peroxide value of unpackaged peanut butter after 150 days of storage was 0.26 g·100 g^−1^, exceeding the limit level of 0.25 g·100 g^−1^, and further increased to 0.83 g·100 g^−1^ after 300 days, indicating a sharp decline in edible quality. The peroxide value of peanut butter packaged with the LDPE film was 0.25 g·100 g^−1^ after 150 days of storage and reached 0.68 g·100 g^−1^ after 300 days. The peroxide value of peanut butter packaged with the PBAT/TPS/GSE-0 film reached 0.25 g·100 g^−1^ after 300 days of storage and increased to 0.59 g·100 g^−1^ after 300 days. The peroxide value of peanut butter packaged with PBAT/TPS/GSE-5 film reached 0.25 g·100 g^−1^ after 300 days of storage. The shelf life of peanut butter packaged with the PBAT/TPS/GSE-5 film was expected to far exceed 300 days, which was approximately twice that of LDPE film packaging. Therefore, the active films prepared in this study significantly delayed the lipid oxidation of peanut butter.

Acid value indicates the degree of rancidity of lipid after slow oxidation, which is usually associated with the hydrolysis of lipid and the oxidation of unsaturated fatty acids to produce acidic substances. As shown in Figure 7b, the acid value of peanut butter increased with storage time. After 300 days of storage, the acid values of unpackaged peanut butter and peanut butter packaged with the LDPE, PBAT/TPS/GSE-0, and PBAT/TPS/GSE-5 films increased from 0.51 mg·g^−1^ to 1.69, 1.60, 1.38, and 1.11 mg·g^−1,^ respectively. The acid values of all samples did not exceed the limit level of 3.0 mg·g^−1^. The low moisture content (1 wt%) of peanut butter results in relatively slow oil hydrolysis. The minimum acid value of peanut butter packaged with the PBAT/TPS/GSE-5 film indicated the improved quality and stability of peanut butter. The migration of GSE from the PBAT/TPS/GSE active films to peanut butter effectively inhibited the oxidation of peanut butter by blocking the chain reaction of lipid oxidation [65].

## 4. Conclusions

This study utilized the industry-representative extrusion blowing technology to fabricate PBAT/TPS/GSE active films incorporating GSE, an agricultural waste, as an active enhancer. The films loaded with GSE demonstrated a progressive intensification of coloration, shifting towards darker hues of yellow and red, and decreased transmittance, which was correlated with higher concentrations of GSE. The addition of GSE inhibited starch plasticization and enhanced the disorder of PBAT molecule arrangement. GSE could primarily interact with starch through hydrogen bonding, with little interaction with PBAT. The incorporation of GSE improved the barrier properties of the films but slightly decreased the mechanical properties. The extrusion-blown PBAT/TPS/GSE active films exhibited effective antioxidant and antimicrobial activities with strong dose dependence. The PBAT/TPS/GSE active films effectively inhibited the oxidative rancidity of packaged peanut butter and extended its shelf life. In addition to packaging easily oxidized peanut butter and similar high-lipid foods, future research will attempt to apply the active films to some foods that are prone to spoilage due to microbial contamination, such as fish, shrimp, meat, and strawberries.

## Figures and Tables

**Table 1:** Color parameters of TPS/PBAT/GSE films.

Film Samples	L*	a*	b*	Δ*E*	T_280_ (%)	T_660_ (%)
PBAT/TPS/GSE-0	91.73 ± 0.10 ^a^	−0.06 ± 0.02 ^d^	5.40 ± 0.07 ^d^	1.13 ± 0.10 ^d^	0.19 ± 0.01 ^a^	11.65 ± 0.09 ^a^
PBAT/TPS/GSE-1	79.27 ± 0.38 ^b^	7.70 ± 0.84 ^c^	17.24 ± 0.30 ^c^	19.27 ± 0.14 ^c^	0.21 ± 0.03 ^a^	9.59 ± 0.11 ^b^
PBAT/TPS/GSE-3	68.81 ± 0.21 ^c^	12.87 ± 0.08 ^b^	26.43 ± 0.10 ^b^	34.06 ± 0.23 ^b^	0.18 ± 0.02 ^a^	5.99 ± 0.20 ^c^
PBAT/TPS/GSE-5	63.36 ± 0.18 ^d^	16.61 ± 0.18 ^a^	29.80 ± 0.18 ^a^	41.30 ± 0.30 ^a^	0.20 ± 0.03 ^a^	5.82 ± 0.17 ^c^

Results are quoted as means ± standard deviation. Different letters within the same column indicate significant differences among the film samples at *p* < 0.05.

**Table 2:** Mechanical and barrier properties of TPS/PBAT/GSE films.

Film Samples	Thickness (μm)	TS (MPa)	EAB (%)	WVP (g·mm·m^−2^·day^−1^·kPa^−1^)	OP (×10^−3^ cm^3^·mm·m^−2^·day^−1^·kPa^−1^)
PBAT/TPS/GSE-0	94.67 ± 2.31 ^a^	15.82 ± 0.65 ^a^	749.33 ± 21.41 ^a^	2.76 ± 0.07 ^a^	4.90 ± 0.18 ^a^
PBAT/TPS/GSE-1	95.67 ± 1.15 ^a^	15.49 ± 0.53 ^a^	732.59 ± 21.30 ^a^	2.61 ± 0.04 ^a^	4.33 ± 0.15 ^b^
PBAT/TPS/GSE-3	92.67 ± 1.53 ^a^	13.82 ± 0.41 ^b^	675.37 ± 18.87 ^b^	2.30 ± 0.05 ^b^	3.87 ± 0.12 ^c^
PBAT/TPS/GSE-5	96.00 ± 2.65 ^a^	13.50 ± 0.52 ^b^	636.53 ± 33.81 ^c^	2.15 ± 0.06 ^c^	3.60 ± 0.14 ^d^

Results are quoted as means ± standard deviation. Different letters within the same column indicate significant differences among the film samples at *p* < 0.05.

## Data Availability

The original contributions presented in the study are included in the article. Further inquiries can be directed to the corresponding author.
